# Development and application of a microarray meter tool to optimize microarray experiments

**DOI:** 10.1186/1756-0500-1-45

**Published:** 2008-07-11

**Authors:** Richard JD Rouse, Katrine Field, Jennifer Lapira, Allen Lee, Ivan Wick, Colleen Eckhardt, C Ramana Bhasker, Laura Soverchia, Gary Hardiman

**Affiliations:** 1 Biomedical Genomics Microarray Facility (BIOGEM), La Jolla CA 92093, USA; 2 HTS Resources, LLC, 11175-A Flintkote Avenue, San Diego, CA 92121, USA; 3 Center for Molecular Genetics, Department of Pediatrics, School of Medicine, University of California, San Diego, La Jolla, CA 92093-0634, USA; 4 Department of Experimental Medicine and Public Health, University of Camerino, 62032 Camerino (MC), Italy; 5 Department of Medicine, University of California San Diego, La Jolla, CA 92093-0724, USA

## Abstract

**Background:**

Successful microarray experimentation requires a complex interplay between the slide chemistry, the printing pins, the nucleic acid probes and targets, and the hybridization milieu. Optimization of these parameters and a careful evaluation of emerging slide chemistries are a prerequisite to any large scale array fabrication effort. We have developed a 'microarray meter' tool which assesses the inherent variations associated with microarray measurement prior to embarking on large scale projects.

**Findings:**

The microarray meter consists of nucleic acid targets (reference and dynamic range control) and probe components. Different plate designs containing identical probe material were formulated to accommodate different robotic and pin designs. We examined the variability in probe quality and quantity (as judged by the amount of DNA printed and remaining post-hybridization) using three robots equipped with capillary printing pins.

**Discussion:**

The generation of microarray data with minimal variation requires consistent quality control of the (DNA microarray) manufacturing and experimental processes. Spot reproducibility is a measure primarily of the variations associated with printing. The microarray meter assesses array quality by measuring the DNA content for every feature. It provides a post-hybridization analysis of array quality by scoring probe performance using three metrics, a) a measure of variability in the signal intensities, b) a measure of the signal dynamic range and c) a measure of variability of the spot morphologies.

## Background

Microarray production efforts require manipulations such as probe desiccation and reconstitution in print buffers, which become increasingly cumbersome with extended library sets. Once a particular print buffer composition has been selected, and the probe library is reconstituted in this solution, switching to an alternate buffer may require further cDNA amplifications or oligonucleotide syntheses to generate additional probes. It is undesirable to waste probe material evaluating immobilization chemistries yet optimization experiments are almost always required [1-4]. As novel slide and print chemistries emerge, in addition to advances in robotic dispensing systems, a thorough evaluation of the best combination of reagents and hardware should be considered before committing the probe collection to the spotting process. This is best achieved through the use of a microarray control set.

A robust microarray control set should a) be easy to implement, b) be applicable to a wide variety of spotting robots, capillary pins and slide chemistries and provide a quality metric for all aspects of a microarray study including array fabrication, c) provide strong signal intensity to every probe on the array thereby facilitating accurate spot-finding, d) be reproducible, facilitating comparison of datasets from different users and laboratories, and e) measure signal intensity over a dynamic range [5].

Researchers typically prepare their own control sets. One example is the AFGC Microarray Control Set [6]. Commercial control sets have also been developed including, The Lucidea Microarray Score Card (Amersham Biosciences) and the SpotReport (Stratagene Inc., La Jolla, CA). These control sets have limited utility for evaluating the parameters in array fabrication, where uniform signal intensity across a given probe concentration is required.

In the present approach we describe a 'microarray meter' which addresses the issues (a-e) outlined above. The probes are engineered with a universal sequence and printed across a defined concentration range, which provides a measure of the amount of DNA required with specific slide chemistries. Two fluorescent labeled targets are hybridized concurrently, a homogenous universal reference labeled with Cy3 and a pool of Cy5 labeled *B. subtilis *mRNAs, which serve as a control to measure signal intensity across a dynamic range. The reference allows assessment of spot detection and feature quality control, whereas the bacterial sequences monitor experimental dynamic range. In this study we applied the microarray meter tool to monitor the efficiency of array fabrication for three commercial microarray spotting robots paired with different capillary pin combinations. The microarray meter tool also permitted an evaluation of different slide and hybridization chemistries, further optimizing experimental conditions prior to the fabrication of high density arrays.

### Development of the microarray meter targets and probes

**Figure 1:**
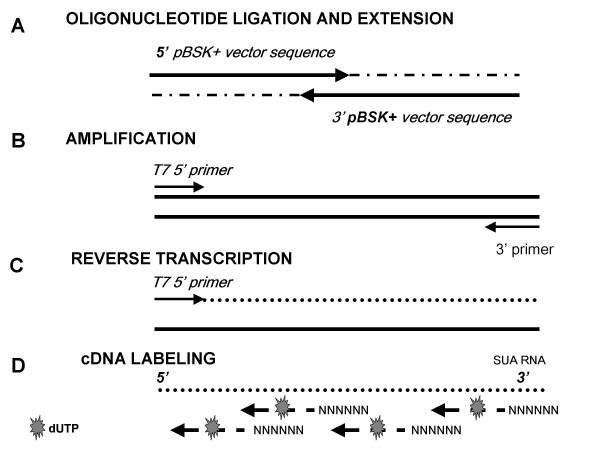
**Schematic of Synthetic Universal Amplicon (SUA) construction**. (a) An extension reaction using two oligonucleotides was used to generate a double stranded DNA product. (b) This product served as template for PCR amplification to attach a T7 RNA promoter sequence. (c) The 127 bp SUA was transcribed *in vitro *to RNA. (d) The RNA product was subsequently reverse transcribed into a fluorescent single stranded cDNA target.

The microarray meter consists of nucleic acid targets (reference and dynamic range control) and probe components. The first target component is a homogenous Synthetic Universal Amplicon (SUA) reference target, created as outlined in Figure 1. The second component comprises a series of dynamic range controls whose sequences are derived from *Bacillus subtilis*. These bacterial sequences were transcribed *in vitro *and each individually labeled with Cy5 (Table 1). After labeling, each of the dynamic range control sequences were individually pooled at defined concentrations (Table 2). The Cy5 dynamic range control and Cy3 SUA targets were co-hybridized to all arrays.

**Table 1:** Analysis of the microarray meter targets.

**Control**	**OD DNA A260**	**OD dye A650/550**	**DNA ng/ul**	**DNA pmol/ul**	**DYE pmol/ul**	**Cy/DNA**	**U/DNA**	**% labeling efficiency**
***ybbR***	0.294	0.322	9.692	0.048	1.288	26.76	50.00	53.53
***ybaQ***	0.221	0.162	7.277	0.037	0.648	17.73	65.25	27.18
***ycxA***	0.202	0.220	6.664	0.033	0.878	26.36	64.25	41.03
***ybaS***	0.219	0.223	7.213	0.038	0.891	23.44	69.00	33.97
***ybaF***	0.270	0.240	8.910	0.048	0.960	20.01	59.25	33.78
***ybdO***	0.189	0.201	6.223	0.039	0.806	20.92	38.00	55.05
***ybaC***	0.405	0.396	13.365	0.073	1.584	21.72	57.50	37.78
***yacK***	0.293	0.293	9.653	0.055	1.170	21.18	50.00	42.36
**SUA**	0.168	0.023	5.440	0.214	1.120	5.22	8.25	63.27

The OD DNA and OD DYE fields list optical density data derived from the spectrophotometer for each target.

Mass concentration (ng/ul), DNA molar concentration (pmol/ul), cyanine dye (DYE) molar concentrations (pmol/ul),

Cy/DNA (ratio of the molecular dye and DNA concentration per target), U/DNA (expected amount of labeled uracil per DNA molecule), percentage labeling efficiency (ratio of expected versus measured labeled uridines per DNA molecule) were determined as outlined in the text.

**Table 2:** Microarray meter dynamic range controls

**Clone ID**	**ng/hyb**	**DNA pmol/hyb**	**Cy5 pmol/hyb**
***ybbR***	2.5	1.24E-02	2.96E-01
***ybaQ***	0.5	2.51E-03	3.97E-02
***ycxA***	0.1	5.00E-04	1.18E-02
***ybaS***	0.04	2.11E-04	4.41E-03
***ybaF***	0.02	1.08E-04	1.92E-03
***ybdO***	0.004	2.48E-05	4.62E-04
***ybaC***	0.0002	1.09E-06	2.11E-05
***yacK***	0	0	0

A dilution series was prepared using the mass concentration of the control stock solutions. The amount of each target calculated as ng DNA, pmol DNA and pmol Cy5 per hybridization is listed.

Additionally there are a series of probe components in the microarray meter design corresponding to *B. subtilis *RNA used here as dynamic range controls. The relationship between each printed probe and the corresponding hybridized target is outlined in Table 3. A dilution series was included to test the optimal spotting concentration of the probes, and identify instrument to instrument variation in this regard.

**Table 3:** Microarray meter probes

**spot ID**	**clone ID**	**probe ng**	**target pg**
***1***	*ybbR*	**200**	2500
***2***	*ybaQ*	**200**	500
***3***	*ycxA*	**200**	100
***4***	*ybaS*	**200**	40
***5***	*ybaF*	**200**	20
***6***	*ybdO*	**200**	40
***7***	*ybaC*	**200**	0.20
***8***	*yacK*	**200**	0
***9***	*blank*	**0**	0

***10***	*ybbR*	**20**	2500
***11***	*ybaQ*	**20**	500
***12***	*ycxA*	**20**	100
***13***	*ybaS*	**20**	40
***14***	*ybaF*	**20**	20
***15***	*ybdO*	**20**	40
***16***	*ybaC*	**20**	0.20
***17***	*yacK*	**20**	0
***18***	*blank*	**0**	0

***19***	*ybbR*	**2**	2500
**20**	*ybaQ*	**2**	500
***21***	*ycxA*	**2**	100
***22***	*ybaS*	**2**	40
***23***	*ybaF*	**2**	20
***24***	*ybdO*	**2**	40
***25***	*ybaC*	***2***	0.20
***26***	*yacK*	***2***	0
***27***	*blank*	**0**	0

***28***	*ycxA*	**50**	100
**29**	*ycxA*	**5**	100
**30**	*ycxA*	**0.5**	100
***31***	*ycxA*	**0.05**	100
***32***	*blank*	**0**	0

The relationship between each printed probe and the corresponding hybridized target is outlined.

Serial dilutions were carried out so that each of the eight probes was diluted from DNA stocks at 200, 20 and 2 ng/l.

One of the probes ycxA was also serially diluted to additional concentrations namely, 5 ng/l, 0.5 ng/l and 0.05 ng/l.

The yacK probe was omitted from the microarray meter plate used with the QArrayMini and BioRobotics Microgrid II robots.

More detail on the microarray meter components is provided in Additional file 1.

### Microarray meter analysis of DNA probe variability, signal dynamic range and feature morphology

We examined the variability in probe quality and quantity (as judged by the amount of DNA printed and remaining post-hybridization) on reflective amino silane slides (Amersham Biosciences) using three robots, The Molecular Dynamics GenIII spotter equipped with GenIII capillary printing pins (Amersham Biosciences, Piscataway, NJ), The QArrayMini (Genetix, Boston, MA) equipped with Telechem ChipMaker™ Pins (Sunnyvale, CA) and The BioRobotics MicroGrid II Robot equipped with MicroSpot 10 K pins (Genomic Solutions, Ann Arbor, MI). Signal intensity following hybridization with the SUA target served as a measure of the amount of DNA deposited. As the hybridization, washing and scanning conditions were identical across all three slide types and as this analysis was carried out post-hybridization, an assessment of the quality of cDNA array fabrication, judged via probe performance, was possible.

**Figure 2:**
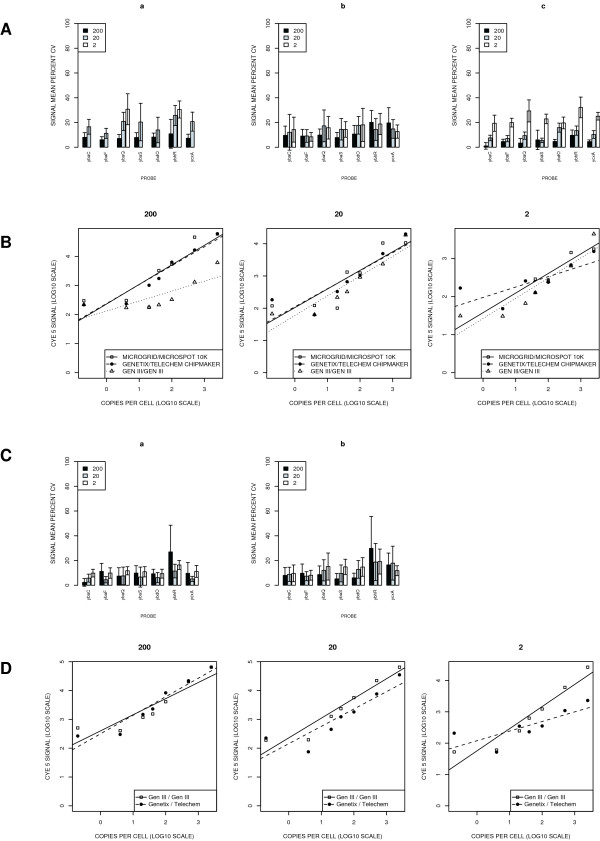
**A: Coefficient of variation (CV) in probe signal intensities on cDNA microarrays**. printed with (a) BioRobotics Microgrid II equipped with MicroSpot 10 K pins, (b) QArrayMini equipped with Telechem ChipMaker Pins, and (c) Molecular Dynamics GenIII spotter. The microarray meter probes were printed from stocks at 200, 20 and 2 ng/μl as described in the text with every capillary pin. **B: Signal dynamic range analysis (cDNA microarrays)**. The signal intensities derived from hybridization of Cy5 labeled dynamic range spikes to increasing concentrations of complementary array probes (2, 20 and 200 ng/μl) was determined and plotted against the abundance of a particular sequence (expressed as an arbitrary copy number) in the hybridization reaction. **C: Coefficient of variation (CV) in probe signal intensities on oligonucleotide microarrays **printed with (a) Molecular Dynamics GenIII spotter and (b) QArrayMini equipped with Telechem ChipMaker Pins. The microarray meter probes were printed from oligonucleotide stocks at 200, 20 and 2 ng/μl as described in the text with every capillary pin. **D: Signal dynamic range analysis (oligonucleotide microarrays)**. The signal intensities derived from hybridization of Cy5 labeled dynamic range spikes to increasing concentrations of complementary array probes was determined and plotted against the abundance of a particular sequence (expressed as an arbitrary copy number) in the hybridization reaction. For the CV analysis presented in **A **and **C**, each robot's 12 pins printed 7 probes at the three different dilutions as 8 replicate spots on a slide. The mean values of the variation observed with each probe across all pins are represented as bars. The error bars denote one standard deviation. Data are plotted on a logarithmic scale in **C **and **D**. Each data point represents the mean of 96 measurements, (each of the 12 pins printed 8 replicate spots per probe per slide).

Every probe was printed with every pin and replicates were spotted. The coefficient of variation (CV) [7] for each of the replicate probes printed from 200, 20 and 2 ng/μl stocks respectively and for each of the 12 pins was calculated and the data is presented in Figure 2A. The CV for each pin across all 7 probes at each dilution range was examined and values were determined to be lowest for the Molecular Dynamics GenIII spotter/GenIII pins (mean, 4.8%), followed by the BioRobotics MicroGrid II/MicroSpot 10 K pins (mean, 7.1%) and the QArrayMini/ChipMaker pins (mean, 11.8%). With decreasing probe concentrations the CV values increased, reflecting greater inconsistency in pin performance at the lower concentrations. At 20 ng/μl CV values were again lowest for the Molecular Dynamics GenIII spotter/GenIII pins (mean, 9.8%), followed by the QArrayMini/ChipMaker pins (mean, 13.8%) and the BioRobotics MicroGrid II/MicroSpot 10 K pins (mean, 20.7%). At the lowest probe concentrations, 2 ng/μl, lowest CVs were observed for the QArrayMini/ChipMaker (mean, 14.5%), followed by the Molecular Dynamics GenIII spotter/GenIII pins (mean, 24.1%) and the BioRobotics MicroGrid II/MicroSpot 10 K pins (mean, 28.4%).

As an additional measure of cDNA array performance we compared signal dynamic range for microarrays fabricated using the three robots. The dilution series for the dynamic range concentrations were prepared by dilution of stock solutions. The Cy5 labeled controls were added per hybridization in defined molar quantities as listed in Table 2. This mirrored the various transcript abundances found within a cell, a feature commonly encountered in a microarray experiment. The resultant Cy5 signal intensities for the microarray meter probes were determined for the different probe concentrations and plotted versus the abundance of a particular dynamic range control (expressed as an arbitrary copy number) in the hybridization reaction (Figure 2B).

This data set revealed a similar performance between the QArrayMini/ChipMaker and the BioRobotics MicroGrid II/MicroSpot 10 K arrays as regards the dynamic range performance of the microarrays. When the probes were printed at higher concentrations (200 ng/μl), the data for these robots followed a linear trend, given the nature of the dilution series for the dynamic range controls. The signal intensities for the corresponding probes on the Molecular Dynamics GenIII printed arrays were lower, and the dynamic range data followed a non-linear profile. The overall probe performance for this robot was reduced at higher DNA concentrations. Microarrays printed using all three robots using the lowest concentration of probe material (2 ng/μl) performed less efficiently due to reduced and variable signal intensities.

The applicability of the microarray meter to 70-mer oligonucleotide arrays was investigated via an analysis of probe performance on reflective amino silane slides using the Molecular Dynamics GenIII spotter/GenIII capillary printing pins and the QArrayMini/Telechem ChipMaker™ Pins. Signal intensity following hybridization with the SUA target served once again as a measure of the amount of DNA deposited. The printing format was similar to that for the cDNA-based arrays, with every probe printed with every pin and with the inclusion of replicate spots. The CV for each of the replicate probes and for each of the 12 pins was calculated and the data is presented in Figure 2C. The corresponding signal dynamic range data is presented in Figure 2D. This revealed comparable probe performance at higher concentrations for both robot and pin combinations, and less variability with the Molecular Dynamics GenIII spotter/GenIII capillary printing pins at lowest probe concentrations. Interestingly the dynamic range data followed a linear profile at higher oligonucleotide concentrations for the Molecular Dynamics GenIII combination.

### Use of the microarray meter to assess feature morphology

**Figure 3:**
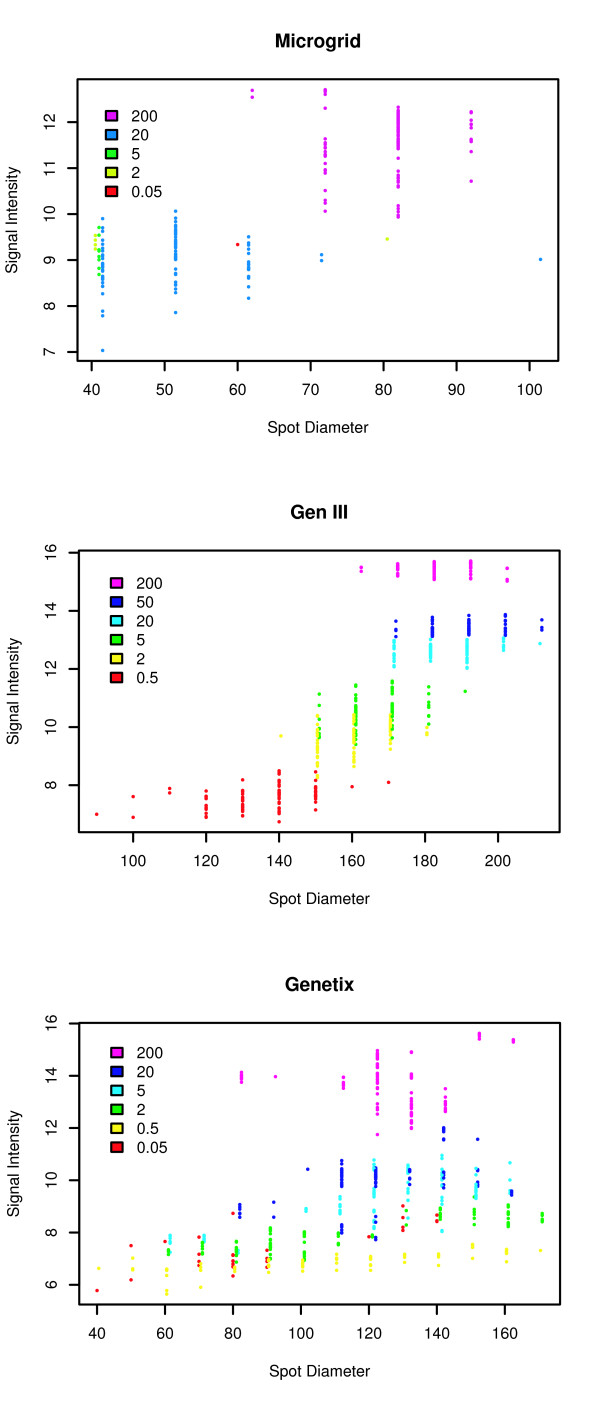
**Analysis of the morphological variability in the array features using the *ycxA *probe**. Plot of signal intensities versus feature diameter. Only probes that passed feature quality control were considered. The data for all three robots is presented.

Analysis of the diameter of the *ycxA *spot following hybridization with the SUA target served as a measure of the efficiency of DNA attachment to the slide surface, and its retention following hybridization (see Additional file 1, supplemental figure S6). In order to fully assess feature morphology an analysis of feature signal intensity versus feature diameter was performed and the data is plotted in Figure 3. Probes that performed poorly were flagged and only those that passed feature quality control were plotted. Compared to the Molecular Dynamics GenIII spotter/GenIII pins and the QArrayMini/ChipMaker pins the BioRobotics MicroGrid II/MicroSpot 10 K pins arrays possessed more flagged features. Spot morphologies with the GenIII spotter/GenIII pins performed best as judged by larger feature sizes and better signal intensities even with lower amounts of probe.

### Use of the microarray meter to compare different slide chemistries and hybridization buffers

The utility of the microarray meter in assessing different print chemistries and hybridization conditions was assessed. The data revealed a similar performance between Amersham reflective Type 7* and Corning Gap II slides as regards the dynamic range performance of the cDNA microarrays (see Additional file 1, supplemental Figure S7). At lower probe concentrations the signal intensities were however significantly higher on the Type 7* surface. Lowest CV values were observed with the Type 7* slides, using DNA probes at a concentration of 200 ng/μl (see Additional file 1, supplemental Figure S8). The microarray meter also determined that an in-house (Type 1) hybridization buffer performed better than a commercial (Type 2) counterpart (see Additional file 1, supplemental Figures S9 and S10).

## Conclusion

Systemic technological biases can confound microarray data interpretation and integration [8-10]. Although different groups have contributed to improving the overall microarray manufacturing process, the microarray meter described in this report is very useful in characterizing the array quality by measuring the DNA content for every array spot [11-14]. This provides a level of confidence for every signal generated and evaluates the performance of both the manufacturing and experimental processes, simultaneously.

Microarrays printed with all three robots using the lowest concentration of the microarray meter probes performed poorly due to reduced signal intensities. This suggests that probes should fall within an experimentally verifiable dilution range with a particular printing instrument to be meaningful to the final analyses. For instance, the microarray meter revealed that probes at 20 ng/μl performed optimally when the Molecular Dynamics GenIII instrument was used to spot arrays. However, this was not the case with the other robots used. Interestingly, less variability in spot diameter was observed using the Molecular Dynamics GenIII instrument even at reduced probe concentrations. The microarray meter has determined that arrays fabricated using this robot coupled with higher DNA concentration performed sub-optimally. Elevated DNA probe concentrations coupled with this robot have a detrimental effect on experimental conditions possibly due to probe saturation. A non-linear profile using the microarray meter was obtained with the 200 ng/μl DNA probe concentration. Consequently empirical determination of the optimal printing conditions is recommended for each robot, pin and slide combination.

There are several means of evaluating microarray quality, prior to hybridization including staining with dimeric cyanine dyes, hybridization with a universal primer or target, hybridization with fluorescently labeled random oligonucleotides or red reflection scanning [15-18]. Unlike some control sets the microarray meter permits visualization of all the probes and not just a subset that serve as fiducial or landmark features. It enables a direct comparison of the different variables associated with array fabrication and experimentation. Furthermore the microarray meter has utility for comparisons of multiple data sets. Many choices exist as regards robotic printers, capillary pins, slide chemistries and hybridization buffers for microarray experimentation. The microarray meter permits a direct comparison of these components, guiding the ultimate choice that is most appropriate to the objective of a particular research program.

In summary the microarray meter tool has been adapted for use with cDNA and oligonucleotide arrays, permitting analyses of the variations introduced by differing combinations of spotting robots and capillary pin.

## Availability

Project Home Page: 

## Authors' contributions

RR and GH conceived the study. RR and LS fabricated the synthetic universal amplicon (SUA), amplified the probes and prepared the fluorescent targets. KF, CE and JL fabricated microarrays and performed hybridizations. CRB, AL and IW provided bioinformatics support. CRB helped draft the manuscript. GH co-ordinated the study and wrote the manuscript. All authors read and approved the final manuscript.

## Conflicts of interests

The authors declare that they have no competing interests.

## Supplementary Material

Additional file 1 The microarray meter consists of nucleic acid targets (reference and dynamic range control) and probe components. A description of the different plate designs formulated to accommodate different robotic and pin designs is provided. Click here for file
