# Supplementary material for: Development and application of a microarray meter tool to optimize microarray experiments
Source: BMC Res Notes. 2008 Jul 11;1:45. doi: 10.1186/1756-0500-1-45 (PMC2535775; doi:10.1186/1756-0500-1-45)
Supplement: Additional file 1 — The microarray meter consists of nucleic acid targets (reference and dynamic range control) and probe components. A description of the different plate designs formulated to accommodate different robotic and pin designs is provided. [file 1756-0500-1-45-S1.pdf]

Supplementary Figures

Supplementary Figure S1

Plate Layout Format for 4x4 pin configuration

A 24 quadrant printing plate layout containing a series of *Bacillus subtilis* DNA sequences, *ybaQ, ybaS, ybhR, ybdO, ybaF, ybaC* and *ycxA*, all present at 200, 20 and 2 ng/µl respectively. In addition *ycxA* was present at 5, 0.5 and 0.05 ng/µl.

Supplementary Figure S2

Plate Layout Format for 1x12 pin configuration

An alternative printing plate containing the same sequences was prepared to accommodate the Molecular Dynamics GenIII spotter which has a 1 x12 proprietary pin format. Each probe was replicated 12 times, with plate columns 1-12 and 13-24 containing replicate probes. All DNA was reconstituted in 50% DMSO.

Supplementary Figure S3

Bacillus subtilis gene sequences used for the microarray meter.

Supplementary Figure S4

Hybridized Microarray Meter Probes

The arrays were hybridized with the SUA and *B. subtilis* targets as described in the text. Relevant portions of the microarray slides are magnified to illustrate the microarray meter probes printed using (A) BioRobotics Microgrid II equipped with MicroSpot 10K pins,

(B) QArrayMini equipped with Telechem ChipMaker Pins and (C) Molecular Dynamics GenIII spotter.

Supplemental Figure S5

Microarray meter oligonucleotide probes derived from *Bacillus subtilis* sequences.

Supplemental Figure S6

Analysis of the morphological variability in the array features using the *ycxA* probe. The coefficient of variation (CV) of the feature diameter on microarrays printed with the BioRobotics Microgrid II, QArrayMini and Molecular Dynamics GenIII spotter was determined and plotted as a function of probe concentration. The *ycxA* probe was printed from six stock probe dilutions ranging in concentration from 200 to 0.05 ng/µl. Each of the 12 pins printed 8 replicate *ycxA* spots per probe per slide. The mean values of the variation observed with each probe concentration across all pins are represented as bars. The error bars denote one standard deviation.

Supplemental Figure S7

Comparison of different slide chemistries by examining signal dynamic range

Examination of signal dynamic range on microarrays printed on Amersham reflective Type 7* and Corning Gap II slides. The signal intensities derived from hybridization of Cy5 labeled dynamic range spikes to increasing concentrations of complementary array probes was determined and plotted against the abundance of a particular sequence (expressed as an arbitrary copy number) in the hybridization reaction. Data are plotted

on a logarithmic scale. Each data point represents the mean of 96 measurements, (each of the 12 pins printed 8 replicate spots per probe per slide).

Supplemental Figure S8

Comparison of different slide chemistries by examining the coefficient of variation of probe intensities

The microarray meter probes were printed from stocks at 200, 20 and 2ng/μl as described in the text with every capillary pin on GapII (A) and Type 7* slides (B). For this CV analysis of all three arraying robots, 12 pins printed 7 probes at the three different concentrations as 8 replicate spots on one slide. The mean values of the variation observed with each probe across all pins are represented as bars. The error bars denote one standard deviation.

Supplemental Figure S9

Comparison of hybridization buffers by examining signal dynamic range

Estimation of a signal dynamic range on microarrays hybridized using two different buffer types (see text for details). Each data point represents the mean of 96 measurements, (each of the 12 pins printed 8 replicate spots per probe per slide).

Supplemental Figure S10

Comparison of hybridization buffers by examining by examining the coefficient of variation of probe intensities

The microarray meter probes were printed from stocks at 200, 20 and 2ng/μl with every capillary pin on Type 7* slides. Hybridization was carried out using either buffer 1 (A) or buffer 2 (B), see text for details. The mean values of the variation observed with each probe across all pins are represented as bars. The error bars denote one standard deviation. For this CV analysis of the hybridization buffers, 12 pins printed 7 probes at the three different concentrations, as 8 replicate spots on one slide.

S1 **Plate Layout Format for 4x4 pin configuration**

	1	2	3	4	5	6	7	8	9	10	11	12	13	14	15	16	17	18	19	20	21	22	23	24
A																								
B																								
C	ybaR 200				ybhR 2				ybaQ 200				ybaS 200				ybdO 200				ybaC 20			
D																								
E																								
F																								
G	ybhR 20				ycxA 20				ybaQ 20				ybaS 20				ybdO 20				ybaC 2			
H																								
I																								
J																								
K	ycxA 200				ycxA 2				ybaQ 2				ybaS 2				ybdO 2				ybaF 20			
L																								
M																								
N																								
O	ycxA 5				ycxA 0.05				ycxA 0.5				ybaF 200				ybaC 200				ybaF 2			
P																								

S2 **Plate Layout Format for 1x12 pin configuration**

	1	2	3	4	5	6	7	8	9	10	11	12	13	14	15	16	17	18	19	20	21	22	23	24
A	ybhR 200	ybhR 200	ybhR 200	ybhR 200	ybhR 200	ybhR 200	ybhR 200	ybhR 200	ybhR 200	ybhR 200	ybhR 200	ybhR 200	yacK 20	yacK 20	yacK 20	yacK 20	yacK 20	yacK 20	yacK 20	yacK 20	yacK 20	yacK 20	yacK 20	yacK 20
B	ybaQ 200	ybaQ 200	ybaQ 200	ybaQ 200	ybaQ 200	ybaQ 200	ybaQ 200	ybaQ 200	ybaQ 200	ybaQ 200	ybaQ 200	ybaQ 200												
C	ycxA 200	ycxA 200	ycxA 200	ycxA 200	ycxA 200	ycxA 200	ycxA 200	ycxA 200	ycxA 200	ycxA 200	ycxA 200	ycxA 200	ybhR 2	ybhR 2	ybhR 2	ybhR 2	ybhR 2	ybhR 2	ybhR 2	ybhR 2	ybhR 2	ybhR 2	ybhR 2	ybhR 2
D	ybaS 200	ybaS 200	ybaS 200	ybaS 200	ybaS 200	ybaS 200	ybaS 200	ybaS 200	ybaS 200	ybaS 200	ybaS 200	ybaS 200	ybaQ 2	ybaQ 2	ybaQ 2	ybaQ 2	ybaQ 2	ybaQ 2	ybaQ 2	ybaQ 2	ybaQ 2	ybaQ 2	ybaQ 2	ybaQ 2
E	ybaF 200	ybaF 200	ybaF 200	ybaF 200	ybaF 200	ybaF 200	ybaF 200	ybaF 200	ybaF 200	ybaF 200	ybaF 200	ybaF 200	ycxA 2	ycxA 2	ycxA 2	ycxA 2	ycxA 2	ycxA 2	ycxA 2	ycxA 2	ycxA 2	ycxA 2	ycxA 2	ycxA 2
F	ybdO 200	ybdO 200	ybdO 200	ybdO 200	ybdO 200	ybdO 200	ybdO 200	ybdO 200	ybdO 200	ybdO 200	ybdO 200	ybdO 200	ybaS 2	ybaS 2	ybaS 2	ybaS 2	ybaS 2	ybaS 2	ybaS 2	ybaS 2	ybaS 2	ybaS 2	ybaS 2	ybaS 2
G	ybaC 200	ybaC 200	ybaC 200	ybaC 200	ybaC 200	ybaC 200	ybaC 200	ybaC 200	ybaC 200	ybaC 200	ybaC 200	ybaC 200	ybaF 2	ybaF 2	ybaF 2	ybaF 2	ybaF 2	ybaF 2	ybaF 2	ybaF 2	ybaF 2	ybaF 2	ybaF 2	ybaF 2
H	yacK 200	yacK 200	yacK 200	yacK 200	yacK 200	yacK 200	yacK 200	yacK 200	yacK 200	yacK 200	yacK 200	yacK 200	ybdO 2	ybdO 2	ybdO 2	ybdO 2	ybdO 2	ybdO 2	ybdO 2	ybdO 2	ybdO 2	ybdO 2	ybdO 2	ybdO 2
I													ybaC 2	ybaC 2	ybaC 2	ybaC 2	ybaC 2	ybaC 2	ybaC 2	ybaC 2	ybaC 2	ybaC 2	ybaC 2	ybaC 2
J	ybhR 20	ybhR 20	ybhR 20	ybhR 20	ybhR 20	ybhR 20	ybhR 20	ybhR 20	ybhR 20	ybhR 20	ybhR 20	ybhR 20	yacK 2	yacK 2	yacK 2	yacK 2	yacK 2	yacK 2	yacK 2	yacK 2	yacK 2	yacK 2	yacK 2	yacK 2
K	ybaQ 20	ybaQ 20	ybaQ 20	ybaQ 20	ybaQ 20	ybaQ 20	ybaQ 20	ybaQ 20	ybaQ 20	ybaQ 20	ybaQ 20	ybaQ 20												
L	ycxA 20	ycxA 20	ycxA 20	ycxA 20	ycxA 20	ycxA 20	ycxA 20	ycxA 20	ycxA 20	ycxA 20	ycxA 20	ycxA 20	ycxA 50	ycxA 50	ycxA 50	ycxA 50	ycxA 50	ycxA 50	ycxA 50	ycxA 50	ycxA 50	ycxA 50	ycxA 50	ycxA 50
M	ybaS 20	ybaS 20	ybaS 20	ybaS 20	ybaS 20	ybaS 20	ybaS 20	ybaS 20	ybaS 20	ybaS 20	ybaS 20	ybaS 20	ycxA 5	ycxA 5	ycxA 5	ycxA 5	ycxA 5	ycxA 5	ycxA 5	ycxA 5	ycxA 5	ycxA 5	ycxA 5	ycxA 5
N	ybaF 20	ybaF 20	ybaF 20	ybaF 20	ybaF 20	ybaF 20	ybaF 20	ybaF 20	ybaF 20	ybaF 20	ybaF 20	ybaF 20	ycxA 0.5	ycxA 0.5	ycxA 0.5	ycxA 0.5	ycxA 0.5	ycxA 0.5	ycxA 0.5	ycxA 0.5	ycxA 0.5	ycxA 0.5	ycxA 0.5	ycxA 0.5
O	ybdO 20	ybdO 20	ybdO 20	ybdO 20	ybdO 20	ybdO 20	ybdO 20	ybdO 20	ybdO 20	ybdO 20	ybdO 20	ybdO 20	ycxA 0.05	ycxA 0.05	ycxA 0.05	ycxA 0.05	ycxA 0.05	ycxA 0.05	ycxA 0.05	ycxA 0.05	ycxA 0.05	ycxA 0.05	ycxA 0.05	ycxA 0.05
P	ybaC 20	ybaC 20	ybaC 20	ybaC 20	ybaC 20	ybaC 20	ybaC 20	ybaC 20	ybaC 20	ybaC 20	ybaC 20	ybaC 20												

Supplemental S3
Bacillus subtilis gene sequences used for the microarray meter

YacK

CCTGTCATCTATATTGCNNTTTTGTTGCTCCGGGTACACCGCTCAGAGCGGGGATGGAAA
ACGTCTTGAGAGCAAATACAGGCGGTCTGATTGTTGTTGGATATAATGATAAAGTAAAAG
AAGTGGTGGACGGCGGCTTTCACATAAACACGGCTTTTTCTCCGGCGCATTTATATGAGC
TGGCTAAAATGGATGGAGCGATCATTTTAAGTGATTCTGGTCAAAAGATCCTATACGCGA
ATACTCAGCTGATGCCGGATGCCACAATTTCTTCATCAGAAACAGGAATGCGGCACAGAA
CTGCCGAAAGAGTAGCTAAGCAAACTGGCTGTCTTGTAATCGCCATTTCTGAAAGAAGAA
ATGTCATAACGTTATATCAGGAAAACATGAAGTATACACTAAAAGACATAGGATTTATTT
TAACCAAGGCGAACCAAGCCATTCAAACACTTGAAAAATATAAGACAATCCTCGATAAAA
CGATTAATGCACTGAACGCGTTAGAGTTTGAGGAACTTGTTACCTTCAGTGATGTCTTGT
CTGTCATGCATCGTTATGAAATGGNACTGGGAATCCAAAACGAAANTTATATGTNTATCC
AAAAACTNGGGGACNAAAANGNCTCTTNATCNAACTGCANNNCCTTNGANTTTTTTNCCG
GATTGGAAAAAAAAGNCCCNTTTTTTTTTTTGGGGCCTTTGTAAAANAAAAAAATNAAAA
ANCCGTTTNNTTCCTTTGNAAGANC

ybaF

GGNAGCATGATTATCGGCAAGTATGTCCCGGGGACTTCACTTGTGCACCGGCTTGACCCC
AGAACAAAACTGATCACGATCTTTTTATTTGTCTGCATTGTATTCTTAGCCAATAATGTT
CAGACATATGCGTTGCTTGGTTTATTTACAATTGGCGTCGTTTCTTTAACAAGAGTTCCT
TTCTCTTTTTTAATGAAAGGGCTGAAGCCAATCATCTGGATTGTCCTTTTTACGTTCCTT
CTTCACATTCTTATGACACATGAAGGACCGATTATCTTTCAAATAGGTTTTTTCAAAGTC
TATGAGGGCGGTCTGGTTCAGGGGATATTCATTTCTCTTAGATTCGTCTATTTGATTTTA
ATCACAACCTTATTAACGCTTACGACTACACCGATTGAGATTACCGATGGAATGGAGCAG
CTGCTGAACCCATTGAAAAAATTAAAGCTTCCTGTTCATGAGCTGGCTCTTATGATGTCA
ATTTCTTTACGGTTTATTCCGACACTGATGGAGGAGACAGATAAGATCATGAAGGCTCAG
ATGGCGCGAGGCGTTGATTTTACAAGCGGGCCTGTCAAAGAAAGAGTGAAGGCTATTGTC
CCGCTTCTTGTCCCGCTCTTCGTCAGCGCATTTAAACGCGCCGAAGAGCTTGCGGTGGCA
ATGGAAGCAAGAGGCTATCAAGGCGGAGNAAGGACGTACNAAATATAGAAAGCTTGTATG
GACAGGAAAAGACACTTCTGTCATGGGCANCCTTAANTGGTATTAGCTGGGA

ybbR

GCTGGGCTGTGAAAATTATTGCTTCTGCTTTTCGCGCTCTTGCTTTATGTGGCGGTTAAC
AGCAACCAAGCACCGACTCCAAAAAAACCGGGTGAATCTTTCTTTCCGACATCAACAACT
GATGAAGCAACTCTGACTGATATTCCGGTTAAAGCGTATTATGATGATGAAAATTACGTC
GTGACGGGTGTTCCGCAAACGGTTAATGTCACGATAAAAGGCTCGACAAGCGCCGTGAAA
AAGGCTCGGCAGACCAAAAACTTTGAAATATATGCCGATATGGAACATTTGAAAACCGGC
ACACATAAGGTTGAGCTTAAGGCCAAAAACGTGTCGGATGGGCTGACAATCTCAATTAAT
CCGTCTGTTACGACAGTGACCATTCAGGAACGGACGACCAAAAGTTTTCCCGTAGAAGTG
GAGTACTACAACAAAAGCAAGATGAAAAAAGGCTATTCTCCGGAGCAGCCGATTGTCAGT
CCGAAAAATGTGCAGATTACCGGATCGAAAAATGTGATCGATAATATTTCTCTTGTAAAA
GCTTCAGTGAATTTGGAAAACGCAGATGAAACGATTGAAAAGGAAGCGAAAGTGACTGTC
TATGATAAAGACGGAAACGCGCTTCCTGTCGACGTGGNAGCCCTCGGTCATCAAGATTAC
CGTTCCGGTGACAAGCCCGAGTAAAAAGTGCCCTTTAAATTGAACGGACAGGAAGCCTTC
CTGACGGTGTCAGCATAGCGAATATTGAATCCAGCCCCNGTGAGGTACGGTTTACGGCTC
CAGGATGTGTTGGATTCTCTTGAATTTTTGACGGCGNCANCCTAAATTTA

yabQ

TTCTATACATGCTAGCGATGNCCGGTATGGGANTCTGGCTTGGTGCTTCGCNTGATACAT
ACCGGCTCTTTGTCATTCGTGCCAAAACAGCCAGATGGCTATTATTTATTCATGATATTC
TTTTCTGGATTATGCAAGGGCTGCTTTTCTTTTATGTCTTGCTTCATGTAAATGAGGGAG
AATTCAGGATTTACATCTTTTTAGCGGTTCTGCTGGGCGTTGCGACGTATCAGAGCCTTT
GCAAACGAATCTATATAAAAATACTGAAATTCGTCATTTACCTTGTTGTTTCTGTTTATC
AATTCTTCAAAAAACTCATTCAGCACGTGTTATTTCGTCCTATTGTGTGGACATGCGGAG
CGATCATCTGGCTGGCGGCATTTTTATTCAAGAAAACATACAGCCTGATAGGTTTTCTCC
TGCTGTGTCTATATAAAATAGTCATGGTTCTGTGTTTTCCGATCCGTTTTATCGCGAAAC
AATGTTTGAAACTTCTTCCTGTGAAAATGCGTCTAACTTTTAGACGTTATTTTGAAAAAG
GTGCAGGATTTCTCAAAAAGAAGAAGAAACTATTGATAACCATAAGAACGACCATCACAG
GATCCAAAAAAAAAAAACTCGANAAGCTTGGCGTAATCATGGTCATAGCTGNTTTCCTGT
GTGAAATTGTTATCCGCTCACAATTCCACACAACATACGAGCCGGAAGCCATAAAGTGTA
AACCCTGGGGGGCCTTAATGNNGTGANCTAACTCACATTAATTGCGTTNCGCTNCCTGCC
CGCTTTCAANCGGGAAANCTGTCGNGCCACCTGCATTAATGAACCGCCACCCGCGGGG

ycxA

ATGCGCCGTCTCCCAGGTCGAAATGGTTTGTATTGCTGTTTACGTTTGTTTTCGCCATCG
GAATGAACTCATTCAGAAATTCCTTTCAATTTTTTATGCTGCCAATGGCAGACGCCTTCC
ATGCCGACAGGTCGCTGATTTCGGTTTCTGTCAGCATTTTTATGATCACAACCGGCATCG
TCCAGTTTTTTGTCGGTTTTTTTATCGACCGTTTCAGTGTCAGAAAAATTATGGCGCTGG
GAGCTGTTTGCATCAGCGCAAGCTTTTTGGTGCTTCCTTATTCACCGAATGTTCATGTGT
TTTCCGCCATTTACGGTGTGCTTGGCGGAATCGGGTATTCCTGCGCGGTCGGCGTGACGA
CCCAGTATTTCATCAGCTGTTGGTTTGACACACACAAAGGTCTGGCGCTTGCTATTTTGA
CCAATGCCAACTCTGCGGGCCTGCTGCTTCTCTCGCCCATTTGGGCTGCGGCTCCGTATC
ATGCCGGCTGGCAGAGCACCTATACCATTTTGGGAATCGTCATGGCGGCTGTTCTGCTGC
CGCTCCTCGTCTTTGGGATGAAGCACCCGCCACATGCGCAAGCGGAAACTGTGAAAAAAT
CTTATGATTGGCGAGGGTTTTGGAACGTGATGAAGCAATCCCGCCTCATTCATATCCTGT
ACTTCGGCGTGTTTACTTGCGGATTTACAATGGGAATTATTGATGCTCACCCTCGTCCCG
ATACTGAAGGATGCGCATGTCTCTCATGTCAACGGAATGATGGCCNCGTTCNGGGGCATT
TATCATCATTGGCGGNATTATGGGCGGGCTGGCTGNCAAATCTCCTCGGCAGC

ybaS

CTGGCTTGGACTTTGCTTTTATTCGTTTGCAGGCAGTTTGAGCGCGAACTTTCAATCTTT
GAGACATGCATTGTCTCATCCTCTCCCTATGATTCTTGCACTATTCGTTCTTCATATTTT
CATGCCGCTTTTTGCTTGGGGCAGCGGCCATCTTATATTTAAAGGTGATCCTTTGACGAT
TACCGGTTTAACATTGGCTGTTGTGATTCCTACGGGGATTACAAGCTTGATTTGGGCAGC
GATGTACAAAGGGAATGTCGGTTTGACACTTTCGATTATTTTAGTTGATACTGTGCTGTC
ACCGCTAATCGTACCGCTGAGTCTTTCATTGCTGGCCGGCGCTCAGGTTCACATGGATGT
GTGGGGAATGATGAAAGGTCTGATTGTAATGGTCGTGATTCCTTCTTTTTTGGGCATGCT
GTTTAATCAGATGTCATCTCCAGAAAGAACTGCGTTTGTGAGCAGTGCGCTGTCGCCTTT
TTCAAAGCTTTGCTTGATGGCAGTGATTGCGATTAACAGTTCAGCGATTGCTCCTTATTT
CNAATCCATTGATTTGAAANTTGCAAGNAANNCTGGNACCGNNATCNTTTTCGCGTTAAC
AGGTTNGCCGCTGCTTGGCTGATCGGAAAATGATGAAAGGCGGCAANAGAAATAGTCTCT
CTTATATTNCGGGAGGCATGAGGAATATTANGCCGGCGCAGTTCTTGCCGTTACTTCTTC
CCTCTCAGGTTGCCGTTCNGTTGTGATCGGCATGCTGTTTCACCANATATTGGCTGCTTT
GTTTGGCTATTGCTGAATCGNT

ybdO

NTGAACGGCAATTTATCCGCANTCAGCCTGATTGCACAGAACATATTGAGCAAGAATGCG
CCTGCCAAACGGGAGAATGCGACTGAAAATCCAGCGGCAGTTCTGGAAAAGGCATATAGC
AGGCTTAAGTCACAATCTTCAACTGGGGGAATCAATCAGTTCAATTATTCTAAAACCAGC
GTGTCGGGAAACAGCGGGACGTTCAGCAAAGTGTATCAATCAGCGAATGATCGAACAGTG
ACAGACACAGGGGAAGAAACAGTGATTCAATCCCAAAACCCCTATGAATCTGAGAGTGAT
ATCAGAATCAAAATACTAGATGAAAAATACAGCAGGATGAATGCGATTAATAAAACAAAG
TCTGATCCATTAGGCTATATTAAGGATAGTACCAAATTCNAAGTCCCCNTTTTTCANAAG
TGATCTGTCANCTGCAAAAAAANAGGCCGCTTACNATAATGAAACAAAANGGNTTTTCNA
AGGGAAGGNCCNAAACTNNACCNTCCGGATGCNGGGTTTCGGANTGCCCCTTTCTTGGGG
AANNGGAACCTNAAANTAAAAGGGNTTNCCCNGGGNNAGGTTACCNNANCCTNAAANNTT
NNNTATCNAAANNNTTTNTTTTNCCCAAGGGGANNNNCNGGCNTTTNAACCCCCCNTTNT
NTTTNGGGNGGGGAANCGGGAAAAANANNGGGTTTTTTNCNNNATTTTGNCAGGGTCCCN
NNGGAAAA

ybaC

CNGTTGCTGCTTTTTTTACATGGCGGGCCGGGAACGCCGCAAATCGGATATGTTAGACAT
TATCAAAAAGAGCTGGAACAGTATTTTACAGTAGTTCATTGGGATCAGAGAGGATCGGGG
CTTTCTTATTCTAAGCGAATTTCGCATCACTCTATGACAATAAATCACTTCATTAAAGAT
ACAATCCAAGTCACTCAATGGCTTTTAGCTCATTTTTCAAAATCAAAACTTTACCTAGCC
GGTCATTCTTGGGGATCAATACTGGCGCTTCATGTGCTGCAGCAGCGTCCTGATTTATTT
TACACGTATTATGGAATCAGCCAGGTTGTTAACCCGCAAGATGAAGAATCAACTGCTTAT
CAACATATTCGTGAAATTTCCGAATCAAAAAAAGCCAGCATATTATCTTTCCTTACACGT
TTCATTGGTGCTCCGCCTTGGAAGCAGGATATCCCAGCACCTTATCTATCNGTTTTGTGT
TGAGCTAACCAGGGGAGGATTCACTCACCGTCATCGTCAATCTCTCGCTGTATTATTTCA
ATGCTTNCTGGCAATGAGTATGGGAGTGCGGAACAATGCACAGCTTCCTTAATGGATTGC
GCTTCAGNAAAAACATTTAACCTGNATGANTTTGTACCGGATTNATGCCTTTTCATCCGN
TCCTTCTATTAAAGNNNCCGGGAGGTTTTCATNTCAGGGGAANCANGACCTTAATTGGTN
CCTGNNGAANATATCGAAAACGCATTANCAAGAACTTGNGGCNCCGAAAANNCGCTTGGT
TNNA

S4 Hybridized Microarray Meter Probes

A

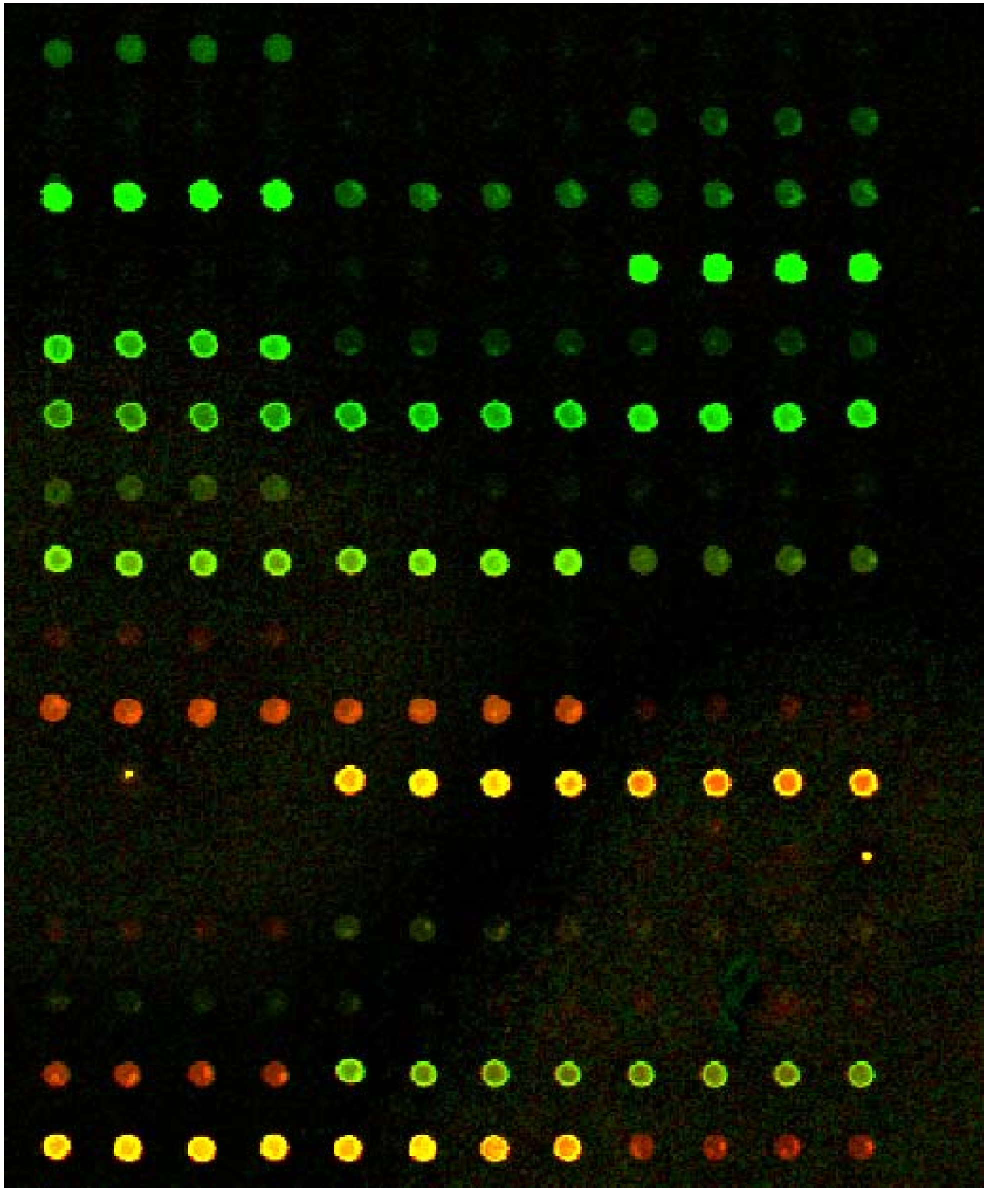

S4 Hybridized Microarray Meter Probes

B

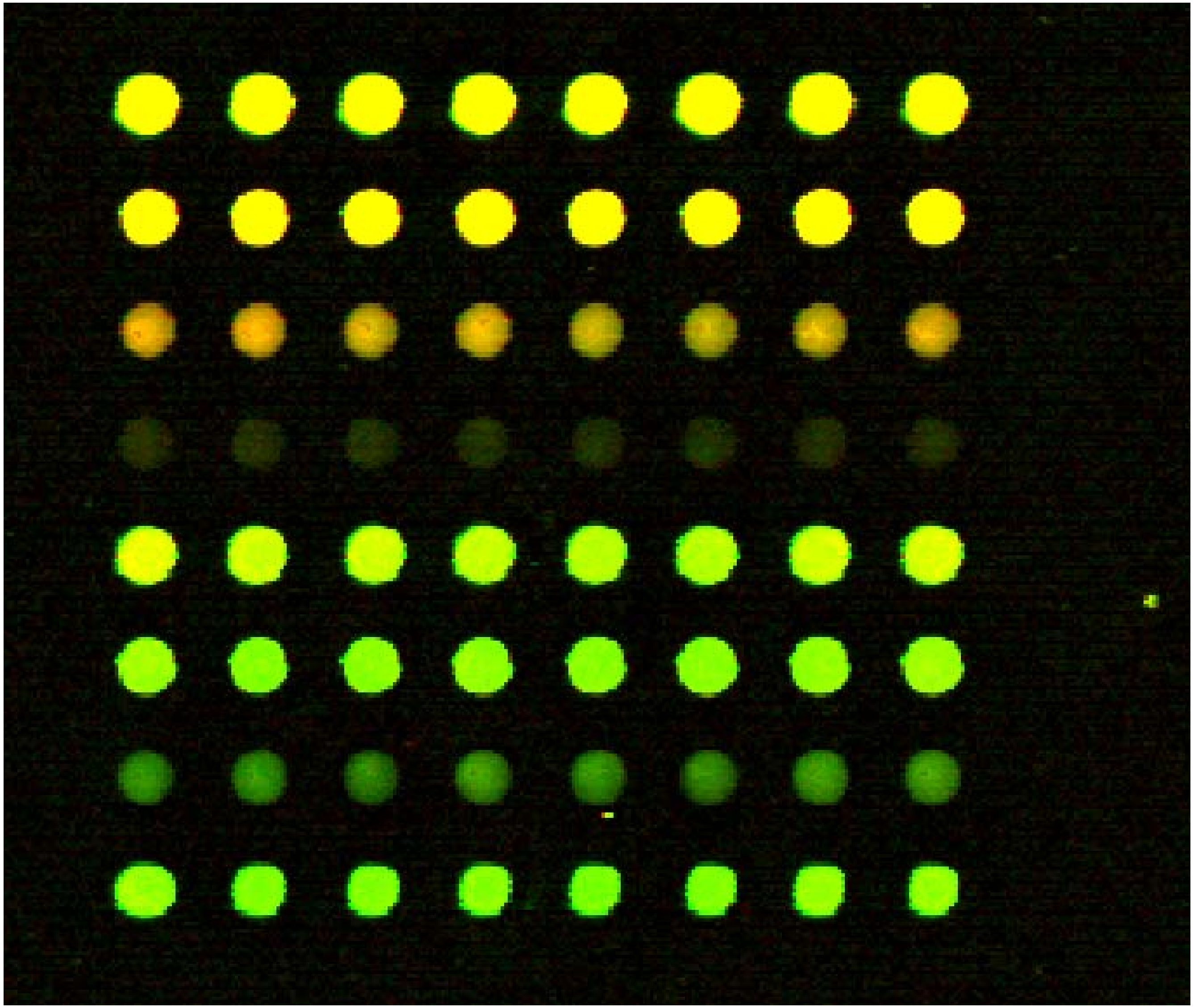

S4 Hybridized Microarray Meter Probes

C

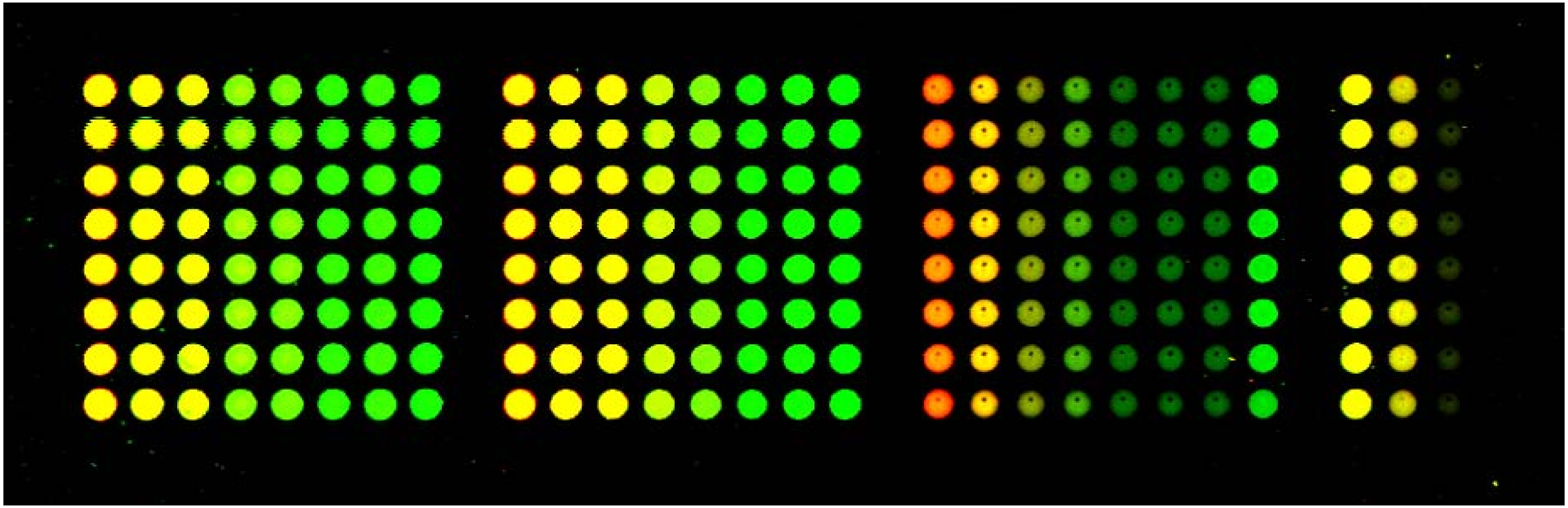

Supplemental S5

Oligonucleotides derived from *Bacillus subtilis* sequences

Seq Name	array meter oligo probe
yabQ	CCCAGTCACGACGTTGCGTACCGCCAGCCAGATGATCGCTCCGCATGTCCACACAATAGGACGAAATAAC
yacK	CCCAGTCACGACGTTGCGTAGAAGAAATTGTGGCATCCGGCATCAGCTGAGTATTCGCGTATAGGATCTT
ybaC	CCCAGTCACGACGTTGCGTAAAGGTGCTGGGATATCCTGCTTCCAAGGCGGAGCACCAATGAAACGTGTA
ybaF	CCCAGTCACGACGTTGCGTATGCTCCATTCCATCGGTAATCTCAATCGGTGTAGTCGTAAGCGTTAATAA
ybaS	CCCAGTCACGACGTTGCGTAGGCGACAGCGCACTGCTCACAAACGCAGTTCTTTCTGGAGATGACATCTG
ybbR	CCCAGTCACGACGTTGCGTAAGTCAGAGTTGCTTCATCAGTTGTTGATGTCGGAAAGAAAGATTCACCCG
ybdO	CCCAGTCACGACGTTGCGTATGATTGATACACTTTGCTGAACGTCCCGCTGTTTCCCGACACGCTGGTTT
ycxA	CCCAGTCACGACGTTGCGTACACGCCGAAGTACAGGATATGAATGAGGCGGGATTGCTTCATCACGTTCC

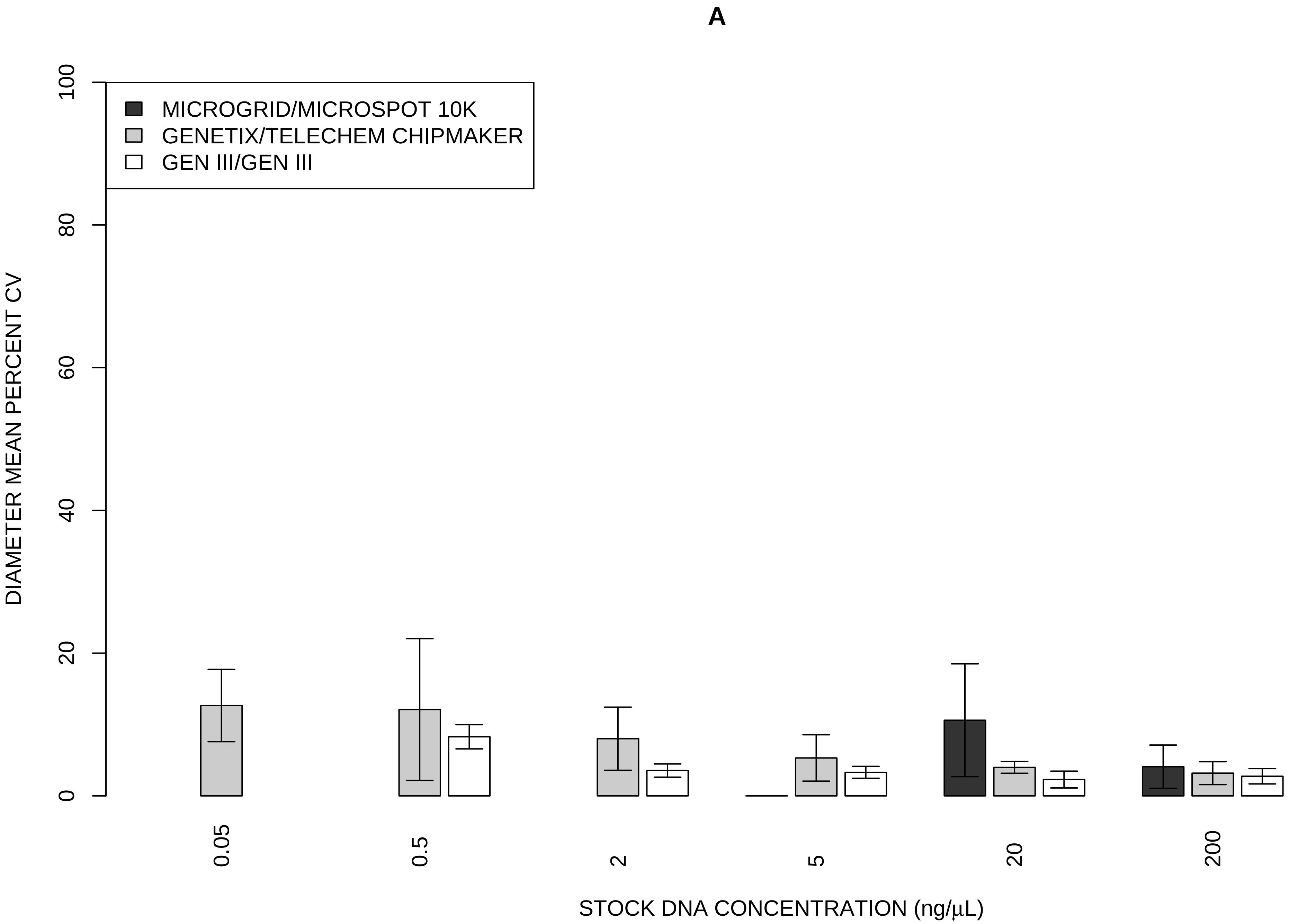

Supplemental S6

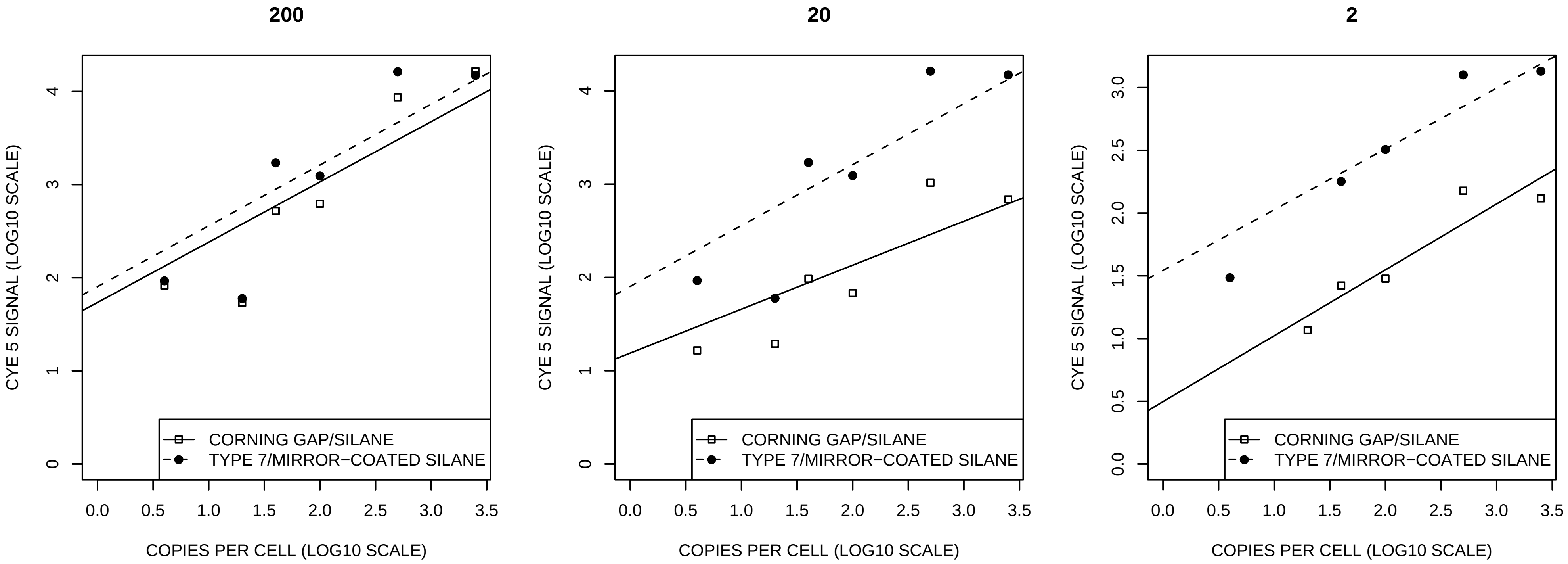

Supplemental S7

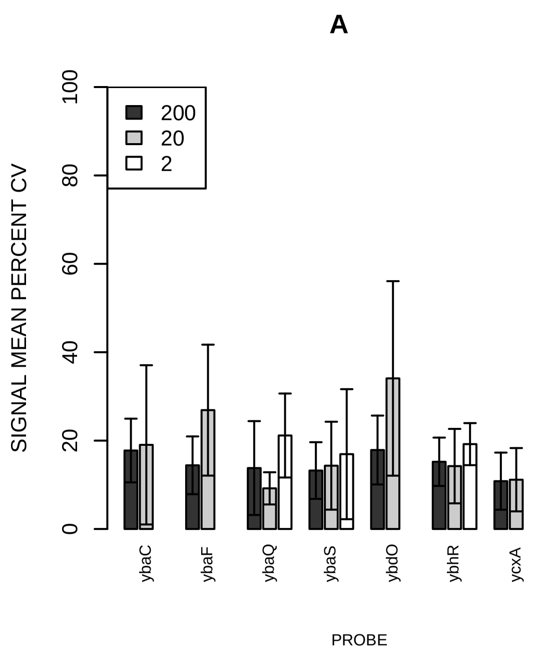

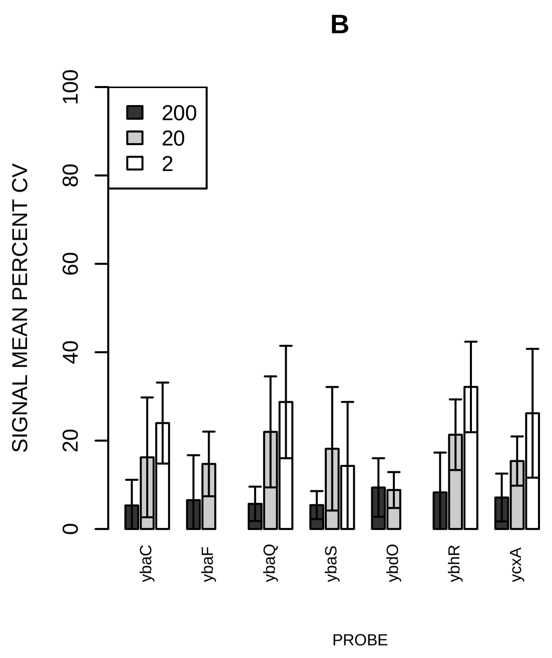

Supplemental S8

Supplemental S9

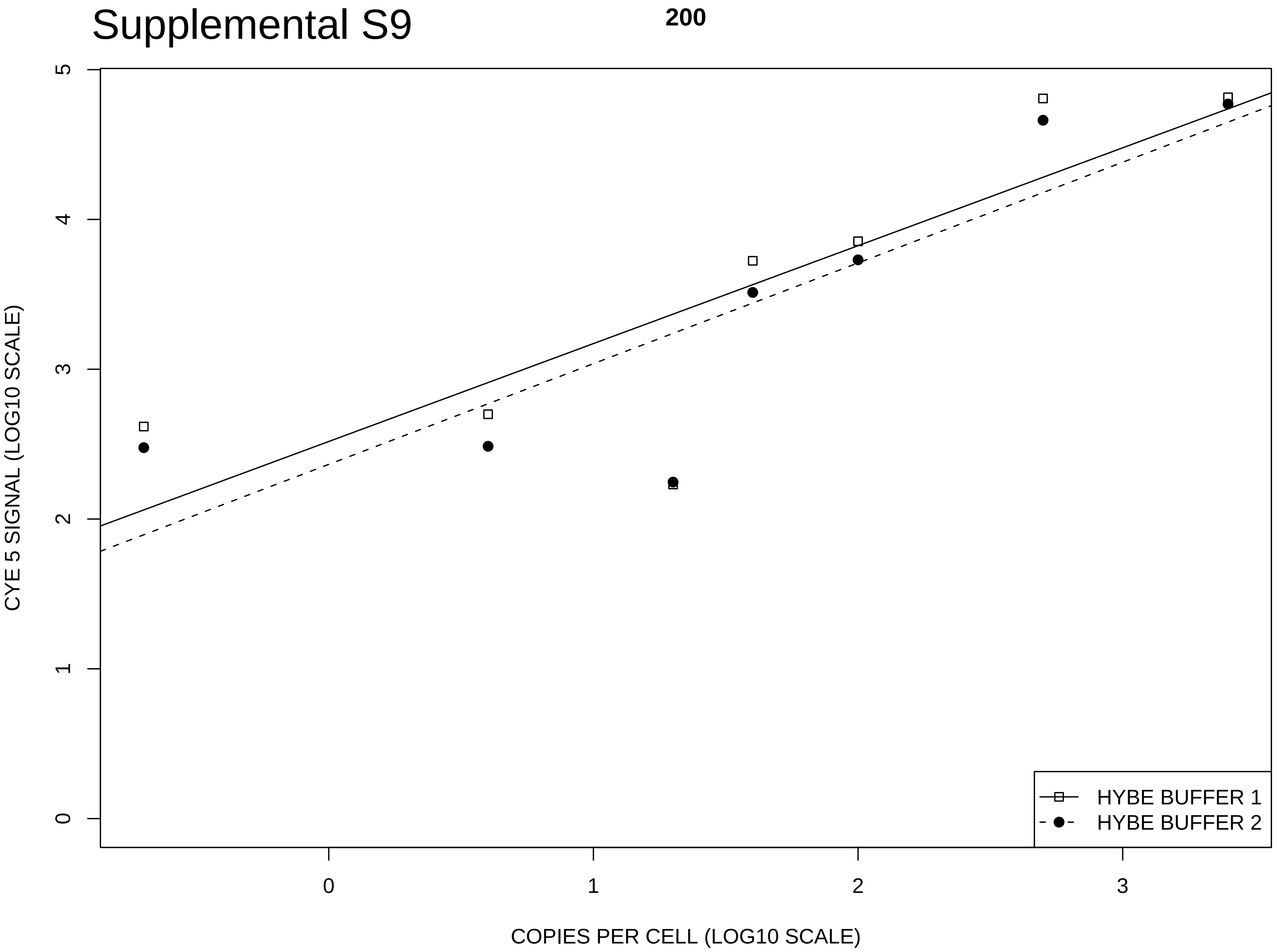

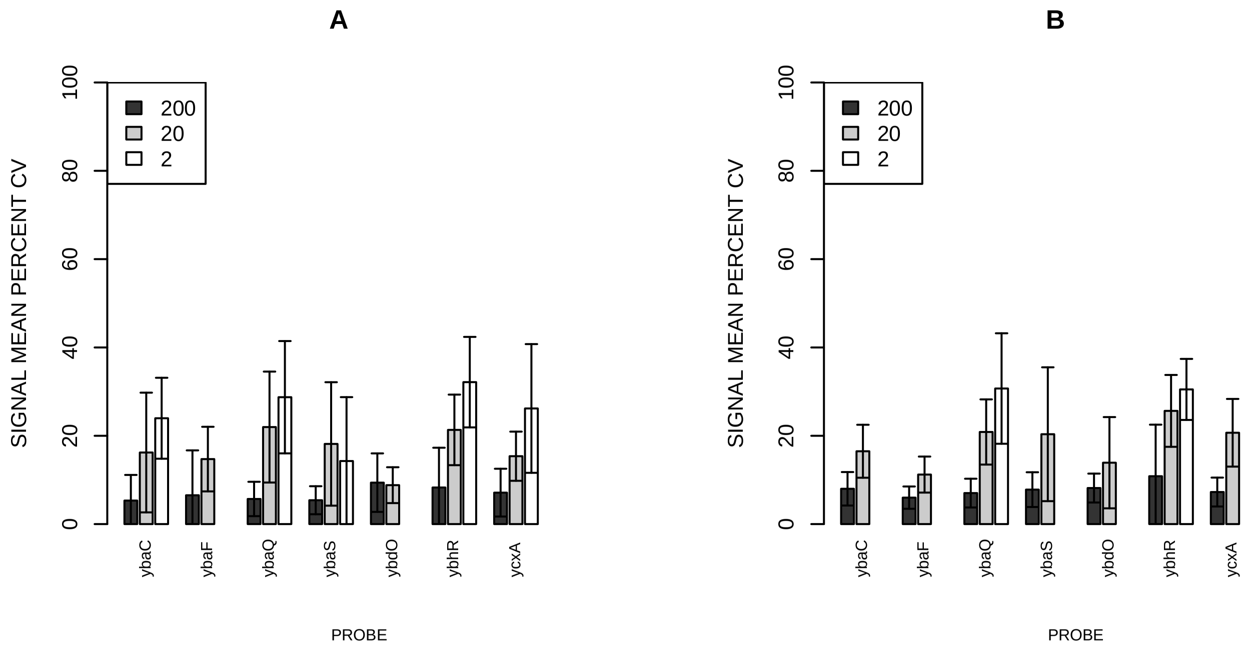

Supplemental S10
